# Oncogenic BRAF Regulates Melanoma Proliferation through the Lineage Specific Factor MITF

**DOI:** 10.1371/journal.pone.0002734

**Published:** 2008-07-16

**Authors:** Claudia Wellbrock, Sareena Rana, Hugh Paterson, Helen Pickersgill, Thijn Brummelkamp, Richard Marais

**Affiliations:** 1 Signal Transduction Team, The Institute of Cancer Research, Cancer Research UK Centre of Cell and Molecular Biology, London, United Kingdom; 2 Oncogene Team, The Institute of Cancer Research, Cancer Research UK Centre of Cell and Molecular Biology, London, United Kingdom; 3 The Whitehead Institute for Biomedical Research, Cambridge, Massachusetts, United States of America; Texas Tech University Health Sciences Center, United States of America

## Abstract

The Microphthalmia-associated transcription factor (MITF) is an important regulator of cell-type specific functions in melanocytic cells. MITF is essential for the survival of pigmented cells, but whereas high levels of MITF drive melanocyte differentiation, lower levels are required to permit proliferation and survival of melanoma cells. MITF is phosphorylated by ERK, and this stimulates its activation, but also targets it for degradation through the ubiquitin-proteosome pathway, coupling MITF degradation to its activation. We have previously shown that because ERK is hyper-activated in melanoma cells in which BRAF is mutated, the MITF protein is constitutively down-regulated. Here we describe another intriguing aspect of MITF regulation by oncogenic BRAF in melanoma cells. We show oncogenic BRAF up-regulates *MITF* transcription through ERK and the transcription factor BRN2 (N-Oct3). In contrast, we show that in melanocytes this pathway does not exist because BRN2 is not expressed, demonstrating that MITF regulation is a newly acquired function of oncogenic BRAF that is not performed by the wild-type protein. Critically, in melanoma cells MITF is required downstream of oncogenic BRAF because it regulates expression of key cell cycle regulatory proteins such as CDK2 and CDK4. Wild-type BRAF does not regulate this pathway in melanocytes. Thus, we show that oncogenic BRAF exerts exquisite control over MITF on two levels. It downregulates the protein by stimulating its degradation, but then counteracts this by increasing transcription through BRN2. Our data suggest that oncogenic BRAF plays a critical role in regulating MITF expression to ensure that its protein levels are compatible with proliferation and survival of melanoma cells. We propose that its ability to appropriate the regulation of this critical factor explains in part why BRAF is such a potent oncogene in melanoma.

## Introduction

Human cancers arise through a multistage process, driven in part by accumulated genetic aberrations that stimulate cancer cell proliferation and survival [1]. Many of these changes, such as the mutations that activate oncogenes, are restricted to particular cell lineages and so are linked to particular types of cancer [2]. An example of this is seen with BRAF, a serine/threonine specific protein kinase that is mutated in 50–70% of spontaneous cutaneous melanomas. The most common mutation in melanoma (over 90% of cases) is a glutamic acid for valine substitution at position 600 (V600E) [3]. ^V600E^BRAF is activated almost 500 fold [4], it transforms immortalised melanocytes [5], and it stimulates proliferation and survival in melanoma cells [6], [7]. ^V600E^BRAF also stimulates melanoma cell invasion *in vitro* and is important for tumour neo-angiogenesis *in vivo* [8]. Furthermore, inhibition of oncogenic BRAF induces tumour shrinkage *in vivo* [7], [9]. These findings show that oncogenic BRAF controls many aspects of melanoma cell biology and yet it is currently unclear why BRAF is such a potent oncogene in the melanocyte lineage.

Another protein that is important in melanoma is the basic helix-loop-helix leucine zipper transcription factor MITF (microphthalmia-associated transcription factor). MITF is considered to be the “master regulator of melanocytes” because it is essential for melanoblast survival and melanocyte lineage commitment. It regulates expression of melanogenic enzymes such as tyrosinase [10], and contributes to melanocyte differentiation by triggering cell cycle exit through induction of the cell cycle inhibitors p16^INK4a^ and p21^Cip^ [11], [12]. We have previously shown that the differentiation functions of MITF occur at high protein concentrations, and accordingly, high levels of MITF are anti-proliferative in melanoma cells [13]. In agreement with this, low levels of MITF protein are found in invasive melanoma cells [14] and are associated with poor prognosis and disease progression in patients [15]–[17]. These data suggest that MITF must be down-regulated for melanoma progression and consistent with this, we have shown that in melanoma cells oncogenic BRAF suppresses MITF protein levels through ERK-mediated phosphorylation and degradation [13].

Despite all the evidence suggesting that MITF must be down-regulated for melanoma progression, MITF expression is essential for proliferation and survival of melanoma cells because it regulates genes such as *CDK2* and *BCL-2* respectively [18], [19]. Furthermore, the *MITF* gene is amplified in 10–15% of melanomas in which BRAF is mutated [20], supporting the view that continued expression of MITF is essential in melanoma cells.

These observations show that the connection between MITF and melanoma development is complex. They also suggest that the interaction between oncogenic BRAF and MITF is more complex than previously reported and this prompted us to examine the interaction between these important proteins in more detail. Here we describe a new aspect of MITF regulation by oncogenic BRAF. We show that BRAF induces MITF transcription through the MEK/ERK cascade and the transcription factor BRN2. Thus, we propose that oncogenic BRAF down-regulates the MITF protein by targeting it for degradation in an ERK-dependent manner, but then counters this by stimulating *MITF* transcription in a BRN2-dependent manner. Through these opposing mechanisms, oncogenic BRAF executes exquisite control over MITF expression, ensuring that the protein levels are permissive for melanoma cell survival and proliferation.

## Results

### MITF expression is dependent on oncogenic BRAF in melanoma cells

**Figure 1:**
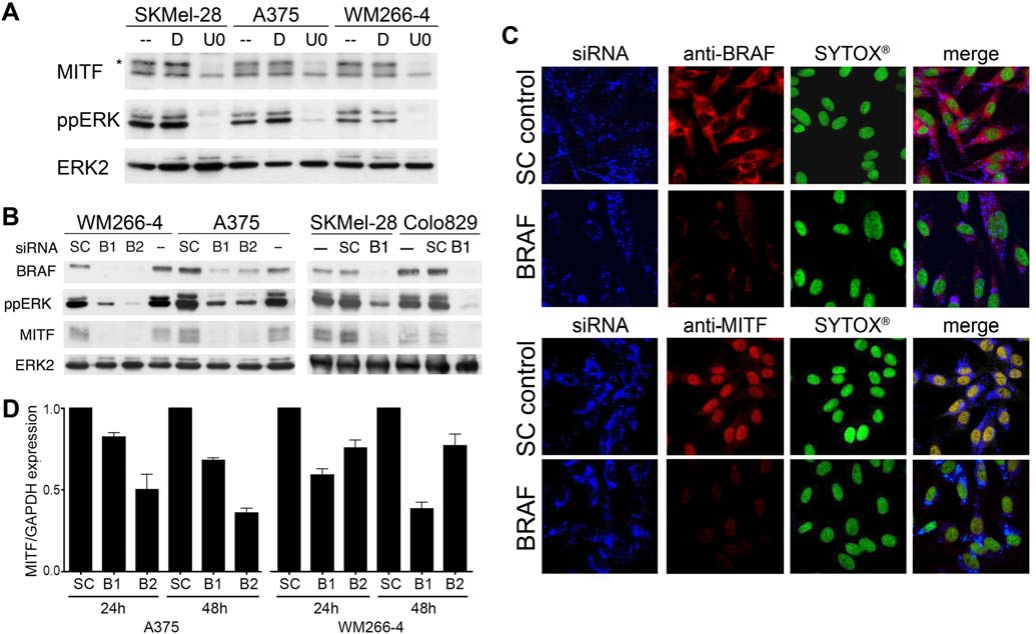
MITF expression requires BRAF in melanoma cells. A) Western blot for MITF, phosphorylated ERK (ppERK) and total ERK2 in untreated (-) melanoma cells or in cells treated with DMSO (D) or U0126 (U0; 10 µM) for 48 h. B) Western blot for BRAF, MITF, ppERK and total ERK2 in melanoma cells 48 h after transfection with control (SC) or BRAF (B1, B2) siRNAs. C) Immunohistochemistry analysis of WM266-4 cells transfected with Alexa-Fluor-647-labelled control (SC) of BRAF siRNA probes (blue). Cells were stained for BRAF or MITF (red) after 48 h and DNA was stained using SYTOX®-green (green). D) Quantitative RT-PCR analysis of MITF in A375 and WM266-4 cells 24 and 48 h after transfection with control (SC) or BRAF (B1, B2) siRNA. MITF expression levels are expressed relative to the GAPDH control.

Previous studies have shown that ERK phosphorylates MITF and targets it for degradation in a proteasome-dependent manner [21], [22]. We have gone on to show that as a consequence of ERK being constitutively activated in melanoma cell lines expressing oncogenic BRAF, MITF protein levels are lower in mutant BRAF melanoma cells than in melanocytes ([13] and see Figure S1). These data suggest that if ERK were inhibited in BRAF mutant melanoma cells, MITF protein levels should increase. We tested this using the MEK inhibitor U0126, which efficiently blocked ERK activity in BRAF mutant melanoma cells (Figure 1A). Notably, this resulted in a significant reduction in the proportion of high molecular weight, phosphorylated forms of MITF in the cells (Figure 1A, see *), but surprisingly long-term MEK/ERK inhibition did not lead to an increase in MITF protein as expected, but rather caused an almost complete loss of MITF expression (Figure 1A). Thus, in apparent contradiction to our previous findings, these data suggest that ERK activation is necessary for MITF expression in melanoma cells expressing oncogenic BRAF.

To corroborate these findings, we used RNA interference (RNAi) to deplete BRAF in melanoma cell lines. This caused the expected reduction in ERK activity (Figure 1B) and in agreement with the U0126 results described above, also caused a reduction in MITF expression (Figure 1B). To ensure the results above were not a post-extraction artefact, we used an immuno-fluorescence approach, depleting BRAF with fluorescently-labelled siRNA oligonucleotides and this also led to a substantial reduction in nuclear MITF staining (Figure 1C). Furthermore, we found that BRAF depletion resulted in reduced *MITF* mRNA expression in melanoma cells (Figure 1D). Thus, although we have shown that oncogenic BRAF/ERK signalling suppresses MITF expression by stimulating its degradation through the ubiquitin pathway [13], the data presented here show that this pathway is also required for MITF mRNA expression in these cells. Together these data suggest that ^V600E^BRAF regulates MITF at both the mRNA level and the protein level, so we further examined the role played by BRAF in the regulation of MITF transcription.

### BRAF regulates MITF transcription through BRN2

**Figure 2:**
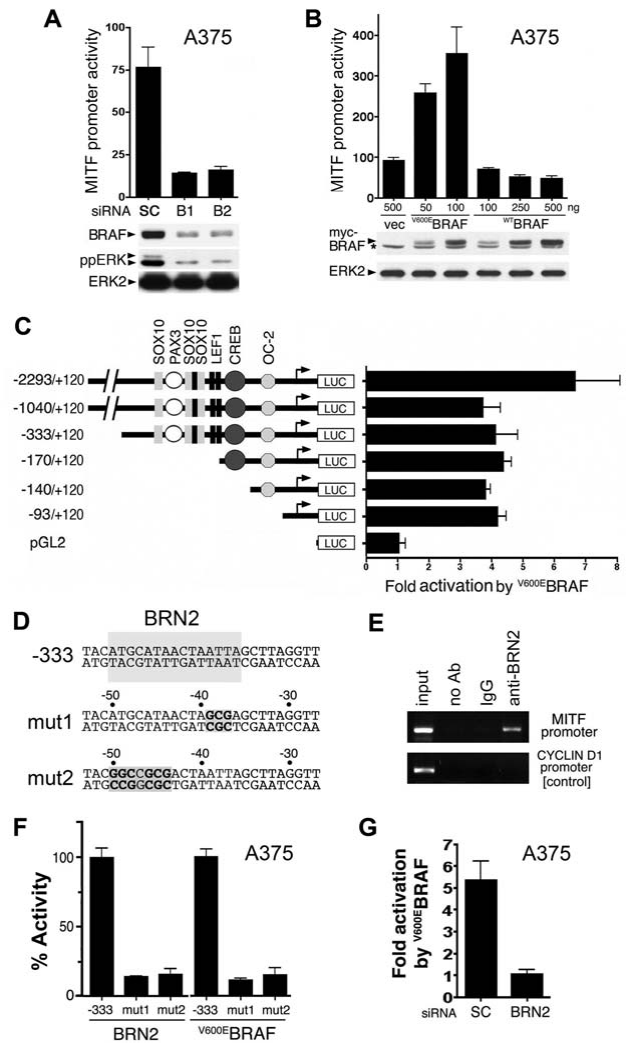
BRAF regulates the MITF promoter through BRN2. A) *MITF* (−2293 to +120) promoter activity in A375 cells transfected with control (SC) or BRAF (B1, B2) siRNA. Extracts were prepared after 48 h and analysed for BRAF and ERK2 (loading control) by Western blotting and for luciferase activity [RLU]. B) *MITF* (−2293 to +120) promoter activity in A375 cells transfected with the indicated amounts (50–500 ng) of myc-tagged ^V600E^BRAF, myc-tagged ^WT^BRAF or an empty vector control (vec). Extracts, prepared 48 h after transfection were analysed for expression of myc-tagged BRAF (*: non-specific band), ERK2 (loading control) by Western blot and for luciferase activity [RLU]. C) *MITF* promoter activity induced by ^V600E^BRAF. Cells were transfected with the indicated promoter fragments and the fold induction stimulated by ^V600E^BRAF is indicated. Binding sites previously identified in the *MITF* promoter are indicated [10]. D) The sequence of the *MITF* promoter from −53 to −27, with the putative BRN2 binding site indicated. The binding site mutations (mut 1 and mut 2) are also shown. E) Chromatin immunoprecipitation (ChIP) assays from A375 cells using BRN2 antibodies, non-specific antibodies (IgG) or no antibody (no Ab). The −170 to +120 region of the *MITF* promoter was amplified, and as a control the *cyclin D1* promoter was also analysed. F) The activity of the −333 luciferase reporter, stimulated by ^V600E^BRAF or BRN2 in A375 cells is shown. The effects of the BRN2 binding site mutations (mut 1, mut 2) are shown, relative (% Activity) to the activity of the non-mutated promoter. G) The activity of the −333 luciferase promoter in A375 cells transfected with control (SC) or BRN2 siRNA is shown. The cells were transfected with ^V600E^BRAF and the reporter construct 24 h after the siRNA had been introduced. Luciferase activity was measured after a further 48 h.

First, we examined *MITF* promoter activity using a luciferase reporter construct. We show that BRAF depletion caused a substantial decrease in the activity of the *MITF* promoter in melanoma cells (Figure 2A). Conversely, ^V600E^BRAF over-expression activated the *MITF* promoter, whereas wild-type BRAF did not (Figure 2B). We mapped the ^V600E^BRAF-responsive element(s) to a promoter fragment of −93 to +120 relative to the transcription start site (Figure 2C). This promoter fragment does not contain any elements previously characterized as being important for *MITF* promoter activity in melanocytic cells ([10] and see Figure 2C). *In silico* analysis of this fragment (http://www.cbrc.jp/research/db/TFSEARCH.html) revealed a putative binding site for the POU-domain transcription factor BRN2 at −50 to −36 relative to the transcription start site (Figure 2D), so we investigated the role of BRN2 in *MITF* regulation in melanoma cells.

BRN2 bound to a fragment (−77 to −20) of the MITF promoter *in vitro*, but not when the putative BRN2 binding site was mutated (data not shown) and chromatin immunoprecipitation (ChIP) assays showed that endogenous BRN2 binds to the proximal region of the *MITF* promoter in melanoma cells (Figure 2E). We used the −333 MITF promoter fragment (Figure 2C) for further analysis because it contains all of the elements previously shown to be relevant to *MITF* regulation in melanocytic cells [10]. BRN2 activated the −333 promoter (Figure 2F), but this activation was significantly reduced when the 5′ or 3′ region the BRN2 element was mutated (mut1, mut2; Figures 2D and 2F). Importantly, the ability of ^V600E^BRAF to activate the MITF promoter was also dramatically reduced when the BRN2 element was mutated (Figure 2F). This suggested that BRN2 acts downstream of BRAF and in agreement with this hypothesis the ^V600E^BRAF-stimulated activation of the MITF promoter was strongly suppressed when endogenous BRN2 was depleted (Figure 2G).

### BRN2 is required for MITF expression in human melanoma cells

**Figure 3:**
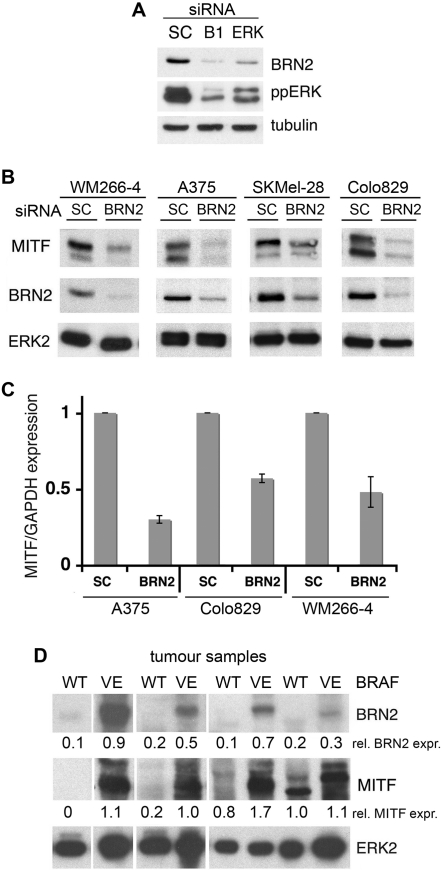
BRN2 is required for MITF expression in melanoma cells. A) Western blot for BRN2, ppERK and tubulin (loading control) in WM266-4 cells treated with control (SC), BRAF or ERK2 siRNA. B) Western blot for MITF, BRN2 and total ERK2 (loading control) in the indicated melanoma cells treated with control (SC) or BRN2 siRNA. C) Quantitative real time PCR of MITF mRNA expression in the indicated melanoma cells treated with control (SC) or BRN2 siRNA. MITF expression was normalised to GAPDH expression and is shown as fold expression in reference to SC transfected cells. Results are for one experiment assayed in triplicate. Similar results were obtained in three independent experiments. D) Western blot for BRN2, MITF and total ERK2 (loading control) in human melanoma samples expressing wild-type BRAF (WT) or ^V600E^BRAF (VE). The expression of BRN2 and MITF relative to ERK2 expression is indicated below the blots.

The data above place BRN2 between ^V600E^BRAF and MITF in melanoma cells and this is consistent with our previous study showing that BRN2 is downstream of ^V600E^BRAF [23]. Here we confirm that BRN2 expression is dependent on ERK signalling downstream of oncogenic BRAF in melanoma cells (Figure 3A). Furthermore, depletion of endogenous BRN2 reduced the levels of endogenous MITF protein in all melanoma cell lines in which BRAF is mutated (Figure 3B). The decrease in MITF protein was due to reduced MITF mRNA expression as determined by quantitative RT-PCR (Figure 3C) and together these findings clearly demonstrate that in melanoma cells BRN2 is required for MITF expression downstream of oncogenic BRAF.

Importantly, we show that BRN2 and MITF expression are strongly correlated with the presence of BRAF mutations in human melanoma samples (Figure 3D). These data link mutant BRAF to BRN2 and MITF expression in clinical samples, suggesting that this pathway is essential for progression of BRAF-driven melanomas.

### MITF regulates the cell cycle downstream of ^V600E^BRAF in melanoma cells

**Figure 4:**
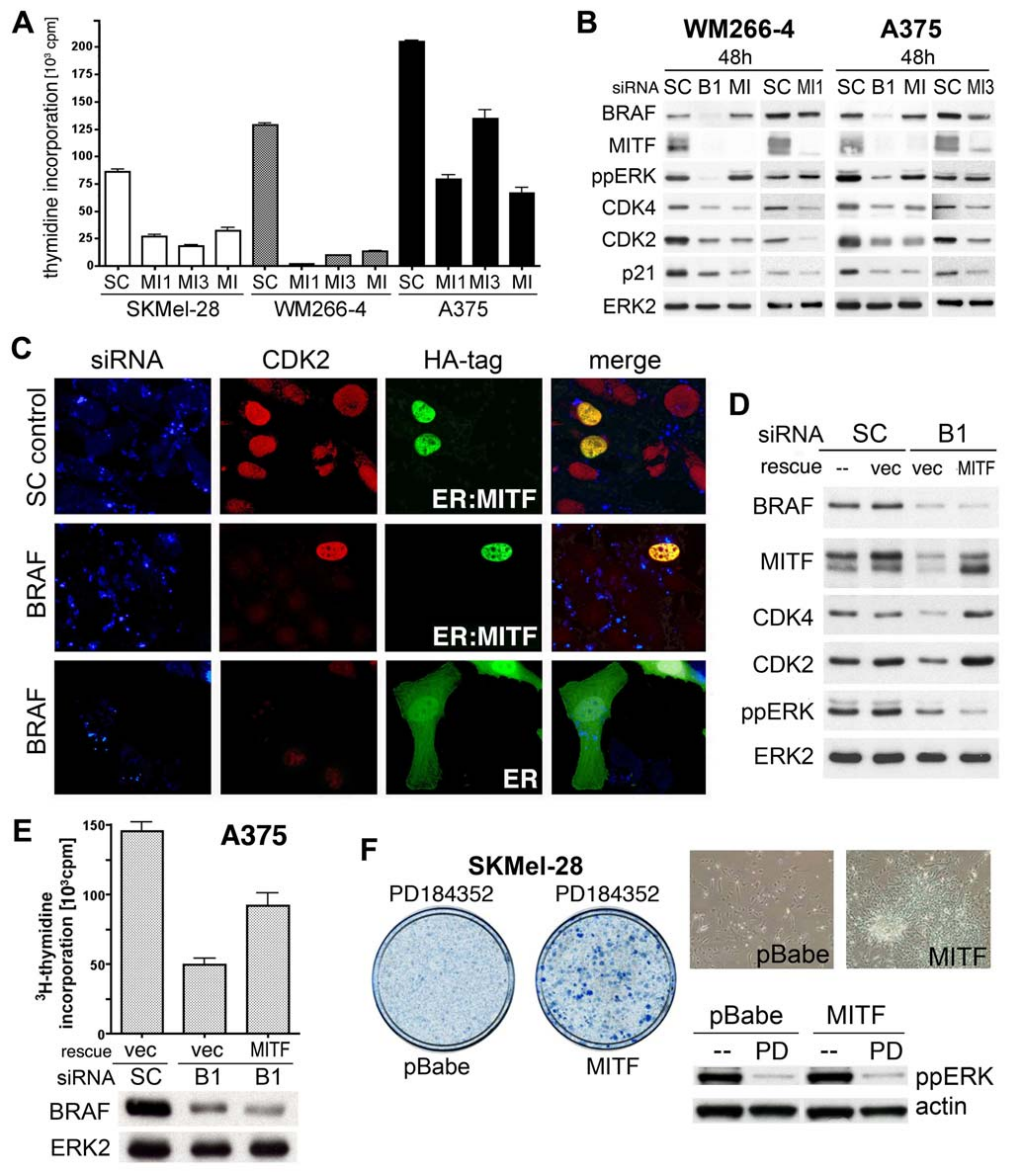
MITF acts downstream of BRAF in melanoma cells. A) DNA synthesis ([^3^H]-thymidine incorporation) in SKMel28, A375 and WM266-4 cells 48–60 h after transfection with control (SC) or MITF (MI, MI1, MI3) siRNA. B) Western blot for BRAF, MITF, ppERK, CDK4, CDK2, p21^Cip^ (p21) and total ERK2 (loading control) in WM266-4 and A375 cells 24 h and 48 h after transfection with control (SC), BRAF (B1) or MITF (MI, MI1, MI3) siRNA. C) Expression of CDK2 in A375 cells transfected with Alexa-Fluor-647-labelled control (SC) or BRAF siRNA (blue). The cells were microinjected with expression constructs for HA-tagged 4OHT-binding domain of the estrogen receptor (ER) or HA-tagged ER∶MITF after 24 hours (green). The cells were treated with 200 nM 4OHT and fixed and stained for CDK2 (red) after a further 40 h. D) Western blot for BRAF, MITF, CDK4, CDK2, ppERK and ERK in A375 cells transfected with control (SC) or BRAF (B1) siRNAs, together with an empty vector (vec) or MITF expression construct (rescue). E) DNA synthesis ([^3^H]-thymidine incorporation) in A375 cells 40 h after transfection with control (SC) or BRAF (B1) siRNA, together with either an empty vector (vec) or MITF expression construct (rescue). The levels of BRAF expressions are shown on a Western blot with ERK2 serving as the loading control. F) Colony formation assay of SKMel28 cells infected with empty vector (pBabe) or MITF expression construct, and treated with 1 µM PD184352 for 4 weeks. Whole plate and high-magnification images are shown. The inhibitory effect of PD184352 on ERK phosphorylation is shown on a Western blot and actin was used as the loading control.

Previous studies have shown that ^V600E^BRAF stimulates melanoma cell proliferation [6], [7] and here we show that MITF is required for proliferation in these cells, because MITF depletion blocks DNA synthesis in BRAF mutant melanoma cells (Figure 4A). Commensurate with their roles in proliferation, depletion of BRAF or MITF caused substantial down-regulation of the cell cycle regulators CDK4, CDK2 and p21^Cip1^ (Figure 4B). CDK2 and p21^Cip1^ are previously identified MITF target genes in melanoma cells [12], [24] and our data suggest that the melanoma susceptibility gene *CDK4* is also an MITF target gene (see also Figure S2).

Importantly, MITF depletion in melanoma cells blocked CDK2, p21^Cip1^ and CDK4 expression even though ERK was not inhibited (Figure 4B). Thus we show that not only is MITF regulated by ERK but also that it functions downstream of this signalling pathway. In agreement with this, CDK2 expression was restored when an oestrogen-regulated version of MITF [12], [13] was expressed and activated in cells in which BRAF was depleted (Figure 4C; see Figure 1A for degree of BRAF depletion). Furthermore, CDK2 and CDK4 expression was also restored when full-length MITF was re-expressed in BRAF depleted cells (Figure 4D and Figure S3) and importantly, this occurred in the absence of ERK reactivation (Figure 4D, lane 4). Critically, MITF re-expression rescued proliferation of melanoma cells when BRAF was depleted using RNAi (Figure 4E), or when MEK was inhibited with PD184352 (Figure 4F). These data show that MITF regulates the expression of critical cell cycle regulatory proteins and stimulates melanoma cell proliferation downstream of BRAF in melanoma cells.

### ^V600E^BRAF, but not ^WT^BRAF regulates MITF expression in melanocytes

**Figure 5:**
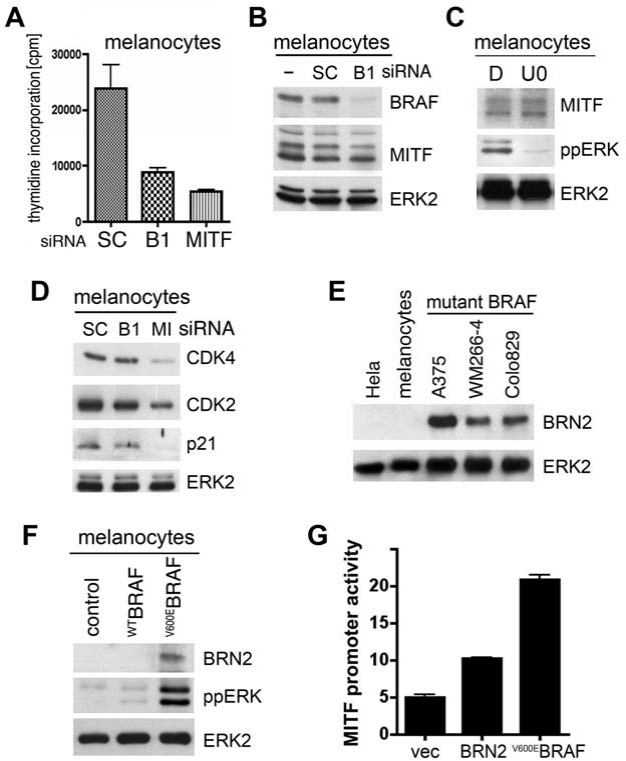
^V600E^BRAF can regulate MITF expression in melanocytes. A) Thymidine incorporation in normal melanocytes 40 h after transfection with control (SC), BRAF (B1) or MITF siRNA. B) Western blot for BRAF, MITF and ERK2 (loading control) in normal human melanocytes 48 h after transfection with control (SC) or BRAF (B1) siRNA. C) Western blot for MITF, ppERK and ERK2 (loading control) in normal human melanocytes 24 h after treatment with DMSO (D) or U0126 (U0; 10 µM). D) Western blot for CDK4, CDK2, p21^Cip^ (p21) and ERK2 (loading control) in human melanocytes 48 h after transfection with control (SC), BRAF (B1) or MITF (MI) siRNA. E) Western blot for BRN2 and total ERK2 (loading control) in Hela cells (negative control), human melanocytes and the indicated melanoma cell lines carrying mutant BRAF. F) Western blot for BRN2, ppERK and total ERK2 (loading control) in melanocytes transfected with empty vector (control), ^WT^BRAF or ^V600E^BRAF. G) *MITF* (−2293 to +120) promoter-reporter (luciferase) activity in melanocytes transfected with an empty vector (vec), or with a BRN2 or ^V600E^BRAF expression construct.

We next examined the interaction between ^WT^BRAF and MITF in normal human melanocytes. As in melanoma cells, BRAF and MITF are both essential for melanocyte proliferation (Figure 5A), but in contrast to melanoma cells, MITF expression does not depend on BRAF expression (Figure 5B) or MEK/ERK signalling (Figure 5C) in melanocytes. This is reflected by a functional independence between BRAF and MITF in melanocytes. Whereas MITF depletion caused down-regulation of CDK4, CDK2 and p21^Cip1^ in melanocytes, these proteins were not down-regulated when ^WT^BRAF was depleted (Figure 5D).

One possible explanation for the lack of MITF regulation by ^WT^BRAF is that unlike melanoma cells, melanocytes do not express BRN2 (Figure 5E). Notably, whereas ^V600E^BRAF can induce BRN2 expression in melanocytes, ^WT^BRAF does not stimulate BRN2 expression in these cells (Figure 5F). Importantly, when introduced into melanocytes, both ^V600E^BRAF and BRN2 can activate the *MITF* promoter (Figure 5G). Thus in melanocytes although ^WT^BRAF does not regulate MITF, ^V600E^BRAF can induce expression of BRN2, allowing it to induce MITF transcription. Notably, the induction of BRN2 through MEK and ERK in melanoma cells appears to be fundamental to the ability of ^V600E^BRAF to regulate MITF in these cells (Figure 3A). Thus, we have established that in addition to regulating MITF at the protein level [13], oncogenic BRAF also regulates MITF at the level of gene transcription in melanocytic cells.

## Discussion

In this study, we show that ^V600E^BRAF regulates the MITF promoter through the octamer-binding transcription factor BRN2 (N-Oct-3). BRN2 is a neuronal-specific protein that is expressed in developing melanoblasts, but which becomes down-regulated as these cells differentiate into melanocytes [25]. Importantly, BRN2 is reactivated in melanoma cells and becomes essential for their proliferation and tumourigenesis [26]. Thus BRN2 re-expression is important for melanoma progression and we have previously shown that BRN2 is a target of oncogenic BRAF in melanoma cells [23]. Here we show that BRN2 expression can also be induced by oncogenic BRAF in melanocytes (Figure 5F). We further demonstrate that BRN2 induces MITF transcription through a binding site located at −50/−36 of the *MITF* promoter, linking MITF expression to oncogenic BRAF through BRN2. Consistent with this, we show that BRN2 and MITF expression occur coincident with oncogenic BRAF in human melanoma samples (Figure 3D). Importantly, we show that wild type BRAF does not induce BRN2 expression in melanocytes, and neither does it stimulate MITF transcription in melanocytes or melanoma cells. Together, these data show that the ability of BRAF to regulate MITF expression is a newly acquired function of the oncogenic protein and that this is mediated through its ability to induce expression of BRN2.

Importantly, we previously reported that ^V600E^BRAF suppresses the endogenous *mitf* promoter in mouse melanocytes [13]. In line with this, we find that ^V600E^BRAF also suppresses the promoter when expressed in mouse melanoma cells (B16 cells, Figure S4). This suggests that the MITF promoter is regulated differently by oncogenic BRAF in mouse and human cells, but it should be mentioned that in contrast to humans, somatic mutations in BRAF do not appear to be a feature of mouse melanomas. Interestingly, we find that like ^V600E^BRAF, BRN2 suppresses the MITF promoter in mouse cells (not shown), suggesting that in mouse cells the presence or absence of certain co-factors might modulate the outcome of the action of BRN2 on the MITF promoter. In summary, we have shown that BRN2 induces MITF mRNA expression in human melanoma cells and that through BRN2, ^V600E^BRAF can stimulate transcription of the *MITF* gene in both melanoma cells and melanocytes.

**Figure 6:**
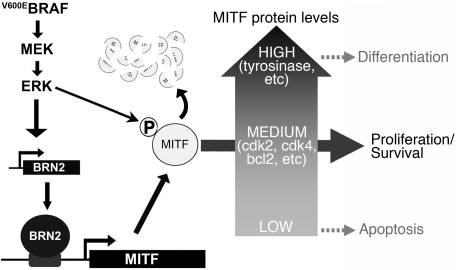
A model of MITF regulation by oncogenic BRAF in melanoma cells. MITF protein levels are critical in melanoma cells. High levels of MITF stimulate differentiation, whereas if the levels are too low, the cells die by apoptosis. Therefore to stimulate proliferation in melanoma cells, MITF protein levels must be constrained to within a narrow range. We propose that ^V600E^BRAF stimulates MITF activation through ERK phosphorylation, but this targets MITF for degradation. This would reduce MITF protein to levels that are below those required for survival and proliferation, so to counter this, ^V600E^BRAF stimulates MITF transcription through up-regulation of BRN2. Thus although MITF is constantly destroyed by proteasome-mediated degradation, its expression persists at a level that is sufficient to maintain expression of cell cycle components such as CDK4 and CDK2 and survival factors such as BCL2, thereby favouring proliferation and survival over differentiation or apoptosis.

It has previously been shown that MITF activation and degradation are coupled because ERK phosphorylation both activates MITF and targets it for degradation through the ubiquitin pathway [21], [22]. Importantly, we demonstrated that because ERK is constitutively activated in BRAF mutant melanoma cells, MITF is constantly degraded ([13] and see Figure S5), a finding that is in agreement with the observation that MITF protein levels are generally lower in BRAF mutant melanoma cell lines than in primary melanocytes (Figure S1). Thus, our demonstration here that ^V600E^BRAF induces *MITF* transcription appears at first to be counter-intuitive. However, MITF plays a critical role in pigment cell behaviour and as previously described, this is partly controlled by the level of MITF in the cells ([13] and see [27]). High levels of MITF trigger cell cycle arrest and differentiation, whereas low levels of activity trigger cell cycle arrest and death (see Figure 6). Thus, MITF has both tumour promoting and tumour inhibitory activities [10] and its ability to control cell fate decisions appears to depend on it levels of expression [13] or possibly its levels of activity [27].

Thus MITF is akin to TGFβ, which, is also either a tumour promoter or a tumour suppressor [28] and we propose that MITF expression/activity must be carefully regulated in melanoma cells to ensure that the levels are not so low as to cause cell death but equally, not so high as to cause cell cycle arrest and differentiation (Figure 6). Our data suggest that oncogenic BRAF plays a key role in regulating MITF to ensure that this is achieved and together with our previous observations, these studies demonstrate that the regulation of MITF by oncogenic BRAF is complex and multifaceted. We posit that because constitutively active ERK is so efficient at down-regulating MITF in BRAF mutant melanoma cells, it is necessary for the cells to rescue MITF expression to ensure that they can continue to proliferate and survive (Figure 6). One strategy that the cells use to achieve this is the activation of the *MITF* promoter by ^V600E^BRAF/ERK/BRN2 as described here. Another strategy appears to be gene amplification, since the *MITF* gene is amplified in approximately 15% of melanoma cases in which *BRAF* is mutated [20]. Notably however, these strategies are not mutually exclusive because although *MITF* is amplified in SKMel-28 cells [20], MITF expression still depends on oncogenic BRAF in these cells (see Figure 1B).

In summary, we show that oncogenic BRAF plays a critical role in regulating MITF expression in melanoma cells, using apparently opposing mechanisms to exquisitely regulate the levels of this critical transcription factor. This ensures that the levels of MITF are optimal for tumour progression and suggests one reason for why BRAF is such a potent oncogene in human melanoma.

## Materials and Methods

### Cell biology approaches

Culture and transfection and in vitro analysis of melanoma cell lines, human melanocytes, stable melanocyte lines and the MITF siRNA MI have been described [5]. Further siRNA oligonucleotides were. For BRAF: B1: AGAAUUGGAUCUGGAUCAU; B2: CAGUCUACAAGGGAAAGUG, for BRN2: BRN2-1: GCGCAGAGCCUGGUGCAGGUU; BRN2-2: CCGCAGCGUCUAACCACUAUU; for MI1: GAACGAAGAAGAAGAUUUA; for MI3: GACCUAACCUGUACAACAA for ERK2: CUCCAAAGCUCUGGACUUAUU. U0126 was from Promega. For thymidine incorporation cells were incubated with ^3^H-thymidine (0.4 µCi/ml) for 4 h before analysis. For the MEK inhibition rescue, 10^6^ SKMel-28 cells were infected with pBABEpuro or pBABEpuro-MITF viruses, selected with 2 µg/ml puromycin for 48 hours and 5×10^4^ of the remaining cells were plated in 10 cm dishes and incubated with 1 µM PD184352 for 4 weeks before being fixed, stained and photographed. The antibodies used were: MITF: C5 and D5 (Neomarkers and generously provided by D. Fisher); BRAF: F-7; Santa Cruz; phospho-ERK: MAPK-YT (Sigma); anti-myc (Abcam); ERK2: C-14 (Santa Cruz); CDK2: M2 (Santa Cruz); CDK4: C-22 (Santa Cruz); BRN2: C-20 (Santa Cruz); p21 (TDL).

### Immunofluorescence

Fixed cells transfected with Alexa-Fluor-647-labelled siRNA probes were incubated with the appropriate primary and Cy3-conjugated secondary antibodies (Dianova). Nuclei were counterstained with SYTOX®-green, and HA-tagged ER or ER:MITF were detected with fluorescein-coupled anti-HA antibody 3F10 (Roche).

### Reverse transcriptate-PCR and Chromatin-immunoprecipitation

RNA was isolated with TRIZOL® and selected genes were amplified by quantitative real time PCR using SYBR green (Qiagen). Primers sequences were *MITF*: CCGTCTCTCACTGGATTGGT, TACTTGGTGGGGTTTTCGAG; *GAPDH*: CAATGACCCCTTCATTGACC, GACAAGCTTCCCGTTCTCAG. Chromatin immunoprecipitation (ChIP) assays were performed with IMGENEX reagents as recommended. Primer sequences were *MITF* promoter: CGTCACTTAAAAAGGTACCTTTATATTTATG, TGTTTTAGCTAGCACCAATCCAGTGAGAGACGG; *cyclin D1* promoter: AACAAAACCAATTAGGAACCTT, ATTTCCTTCATCTTGTCCTTCT.

### Luciferase Reporter Constructs and Assays

BRAF and ^V600E^BRAF expression constructs are described and BRN2 was expressed using pEFPlink.6 [4]. Approximately 2.3 kb of the *MITF* promoter (−2293 to +120) and the various truncated promoters were cloned into pGL2 (Promega). Mutant promoter constructs (mut1, mut2) were generated by PCR directed mutagenesis. Cells were transfected with 0.6 µg of the indicated promoter-reporter constructs, 0.3 µg of BRAF, ^V600E^BRAF or BRN2 expression constructs and 0.3 µg of pSV-ß-Galactosidase (Promega) using Lipofectamine (Gibco). Cells were analysed for luciferase activity after 48 h using RLB buffer (Promega). The data were corrected for b-galactosidase and represent the activity for assays performed in triplicate, with error bars to represent standard deviations from the mean. All experiments were performed a minimum of 3 times.

## Supporting Information

Figure S1 Melanoma cells express lower levels of MITF than melanocytes. Human melanocytes and melanoma cells expressing oncogenic BRAF were analysed for MITF expression. ERK2 served as loading control.
(0.24 MB TIF)

Figure S2 MITF regulates CDK2 and CDK4 transcription. CDK4 expression is regulated by MITF in melanoma cells. (A) RT-PCR for CDK4 and CDK2 (control) in A375 and WM266-4 cells transfected with either control (SC) or MITF (MI) siRNAs. Cells were analysed 24 and 48 hours after transfection and GAPDH serves as a loading control. (B) Real-time RT-PCR for CDK4 in A375 and WM266-4 cells transfected with either control (SC) or MITF (MI) siRNAs. Cells were analysed 24 and 48 hours after transfection. CDK4 expression is shown as fold expression in reference to SC transfected cells and relative to GAPDH expression.
(0.47 MB TIF)

Figure S3 MITF regulates CDK2 and CDK4 transcription downstream of oncogenic BRAF. Quantification of CDK2 and CDK4 expresssion. A375 cells transfected with control (SC) or BRAF (B1) siRNAs, together with an empty vector (vec) or an MITF expression construct (rescue) were analysed for CDK2 and CDK4 and the expression was quantified using ImageQuant (Amersham, GE-Healthcare).
(0.24 MB TIF)

Figure S4 Oncogenic BRAF suppresses the MITF promoter in mouse melanoma cells. V600EBRAF suppresses the MITF promoter in mouse melanoma cells. (A) Luciferase assay for the mouse MITF promoter activity in B16 cells transfected with vector (vec) or V600EBRAF as indicated. The cells were analysed 48 h after transfection. (B) Luciferase assay for the human MITF promoter activity in B16 cells transfected with vector (vec) or V600EBRAF as indicated.
(0.35 MB TIF)

Figure S5 Oncogenic BRAF suppresses MITF protein expression. Oncogenic BRAF suppresses MITF protein levels. Western blot of human melanocytes transfected with V600EBRAF or an empty vector. Cells were analysed for myc-tagged V600EBRAF, MITF and ppERK. ERK2 was used as loading control.
(0.21 MB TIF)
