# Supplementary figures and images for: Oncogenic BRAF Regulates Melanoma Proliferation through the Lineage Specific Factor MITF
Source: PLoS One. 2008 Jul 16;3(7):e2734. doi: 10.1371/journal.pone.0002734 (PMC2444043; doi:10.1371/journal.pone.0002734)

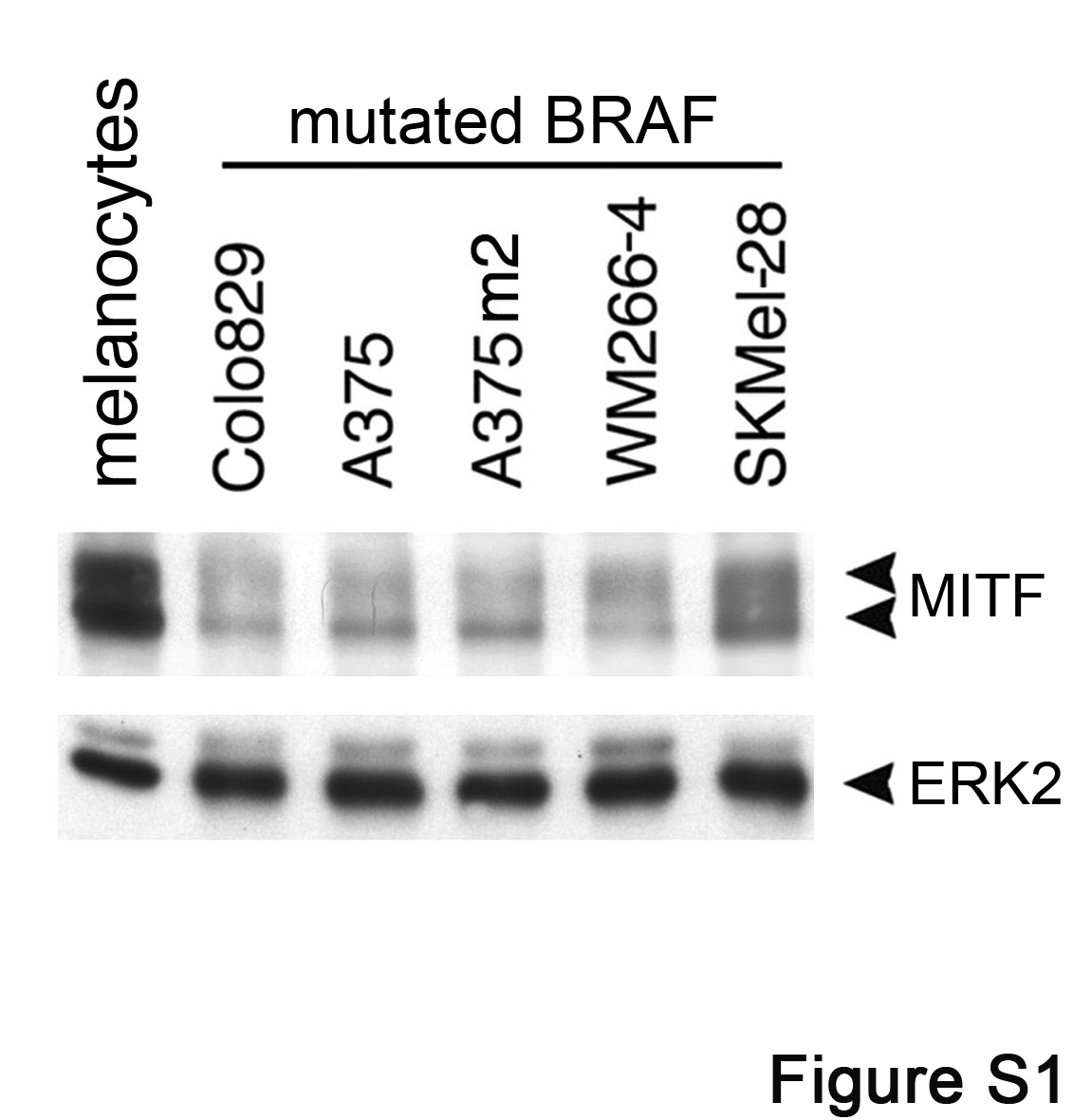

Supplement: Figure S1 — Melanoma cells express lower levels of MITF than melanocytes. Human melanocytes and melanoma cells expressing oncogenic BRAF were analysed for MITF expression. ERK2 served as loading control. (0.24 MB TIF) [file pone.0002734.s001.tif]

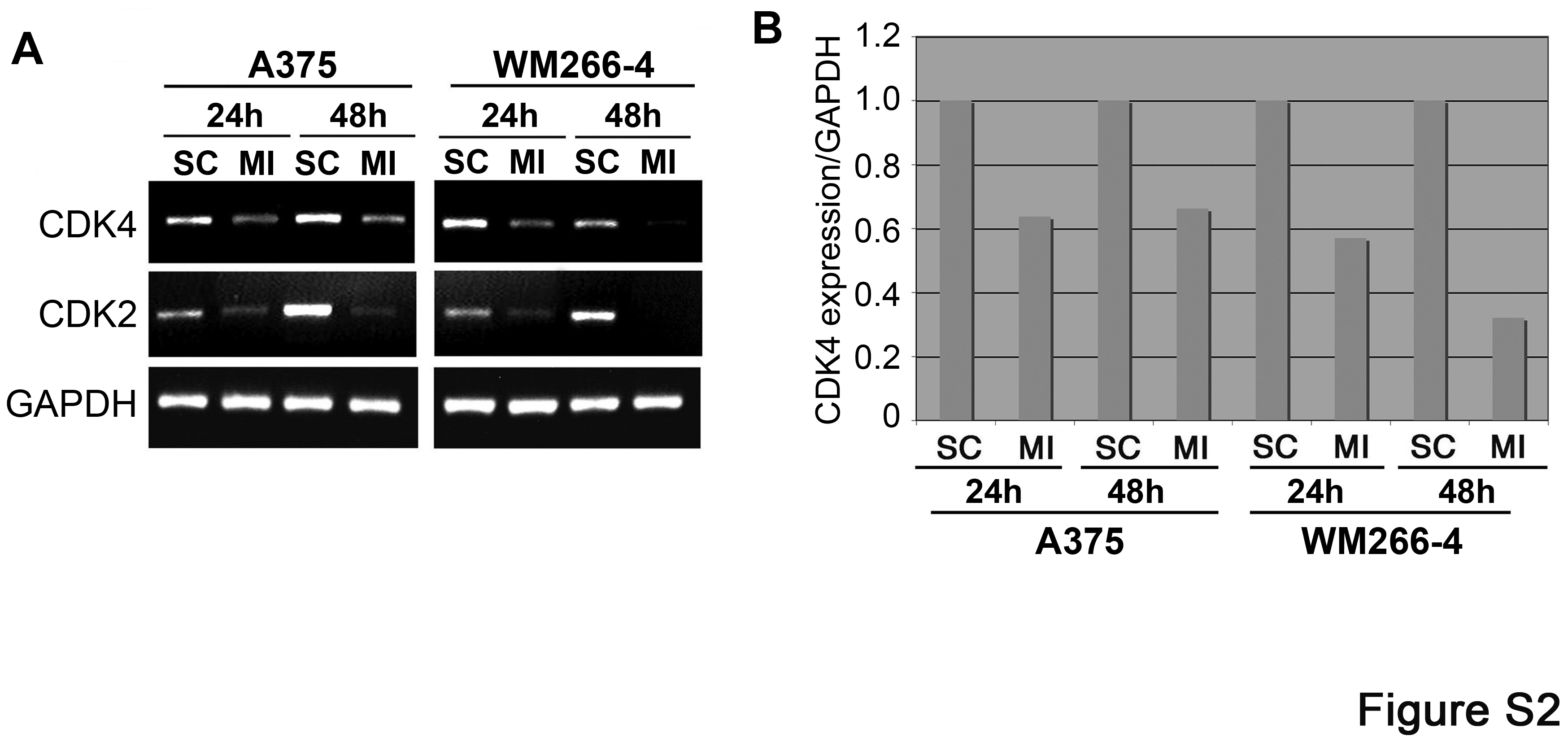

Supplement: Figure S2 — MITF regulates CDK2 and CDK4 transcription. CDK4 expression is regulated by MITF in melanoma cells. (A) RT-PCR for CDK4 and CDK2 (control) in A375 and WM266-4 cells transfected with either control (SC) or MITF (MI) siRNAs. Cells were analysed 24 and 48 hours after transfection and GAPDH serves as a loading control. (B) Real-time RT-PCR for CDK4 in A375 and WM266-4 cells transfected with either control (SC) or MITF (MI) siRNAs. Cells were analysed 24 and 48 hours after transfection. CDK4 expression is shown as fold expression in reference to SC transfected cells and relative to GAPDH expression. (0.47 MB TIF) [file pone.0002734.s002.tif]

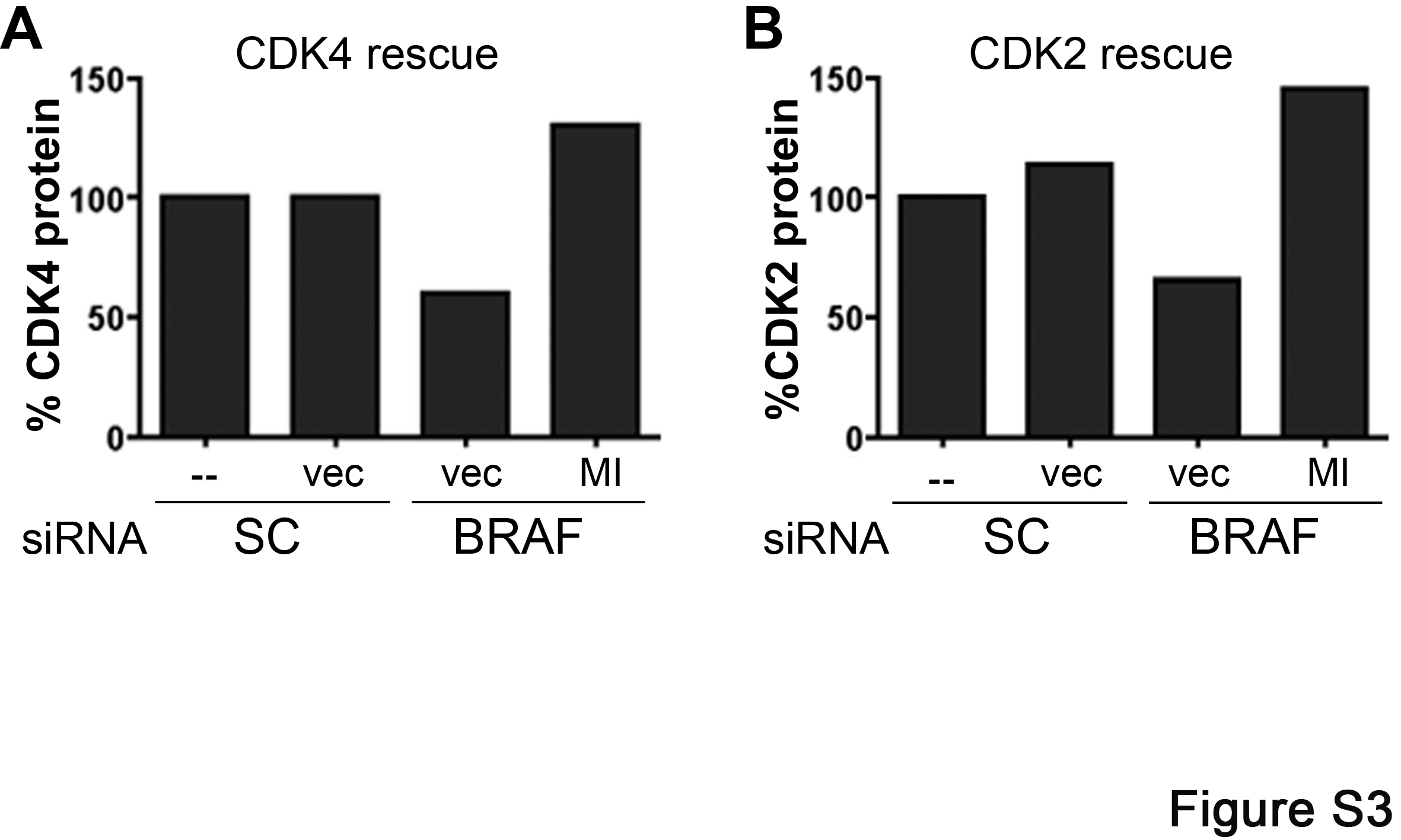

Supplement: Figure S3 — MITF regulates CDK2 and CDK4 transcription downstream of oncogenic BRAF. Quantification of CDK2 and CDK4 expresssion. A375 cells transfected with control (SC) or BRAF (B1) siRNAs, together with an empty vector (vec) or an MITF expression construct (rescue) were analysed for CDK2 and CDK4 and the expression was quantified using ImageQuant (Amersham, GE-Healthcare). (0.24 MB TIF) [file pone.0002734.s003.tif]

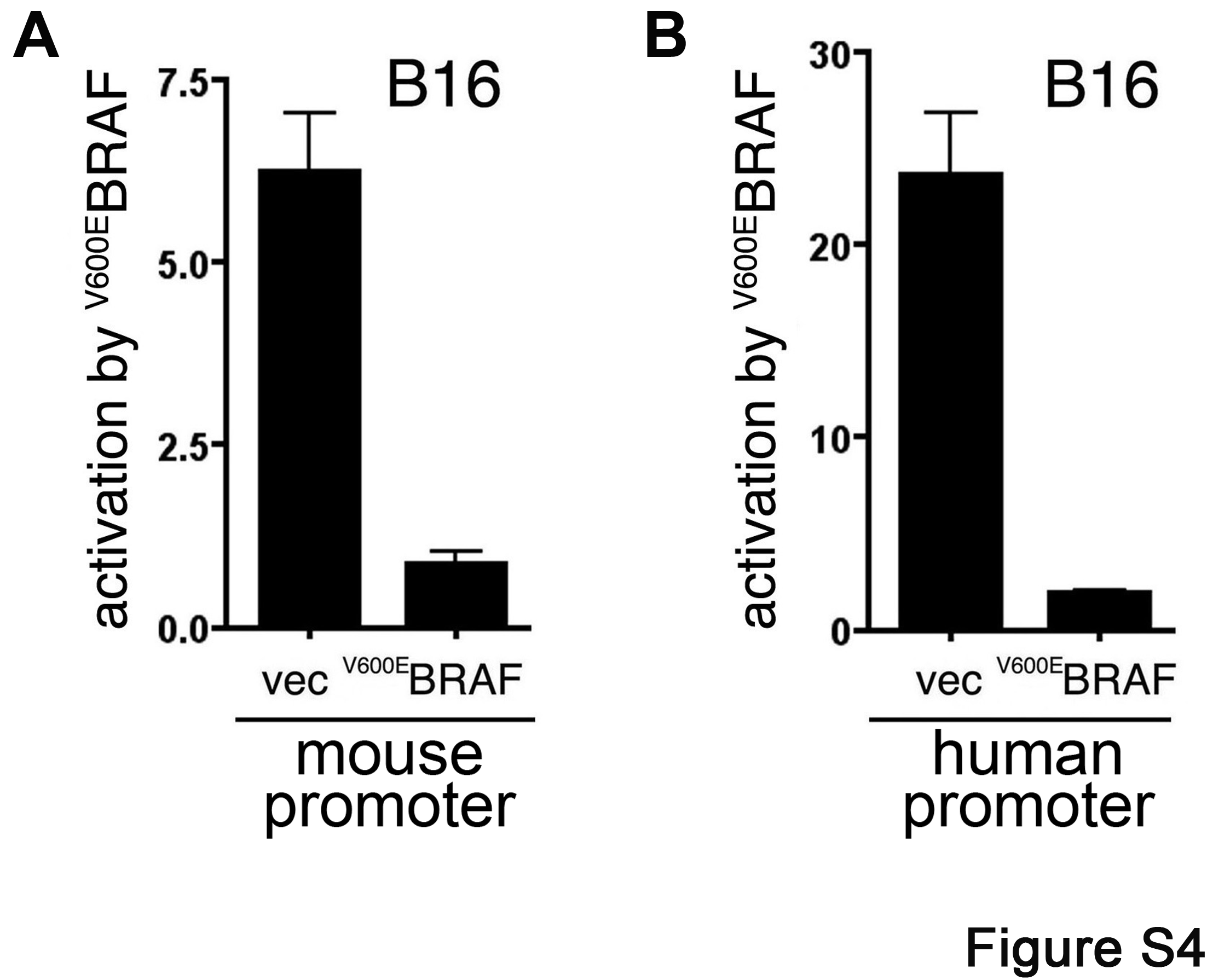

Supplement: Figure S4 — Oncogenic BRAF suppresses the MITF promoter in mouse melanoma cells. V600EBRAF suppresses the MITF promoter in mouse melanoma cells. (A) Luciferase assay for the mouse MITF promoter activity in B16 cells transfected with vector (vec) or V600EBRAF as indicated. The cells were analysed 48 h after transfection. (B) Luciferase assay for the human MITF promoter activity in B16 cells transfected with vector (vec) or V600EBRAF as indicated. (0.35 MB TIF) [file pone.0002734.s004.tif]

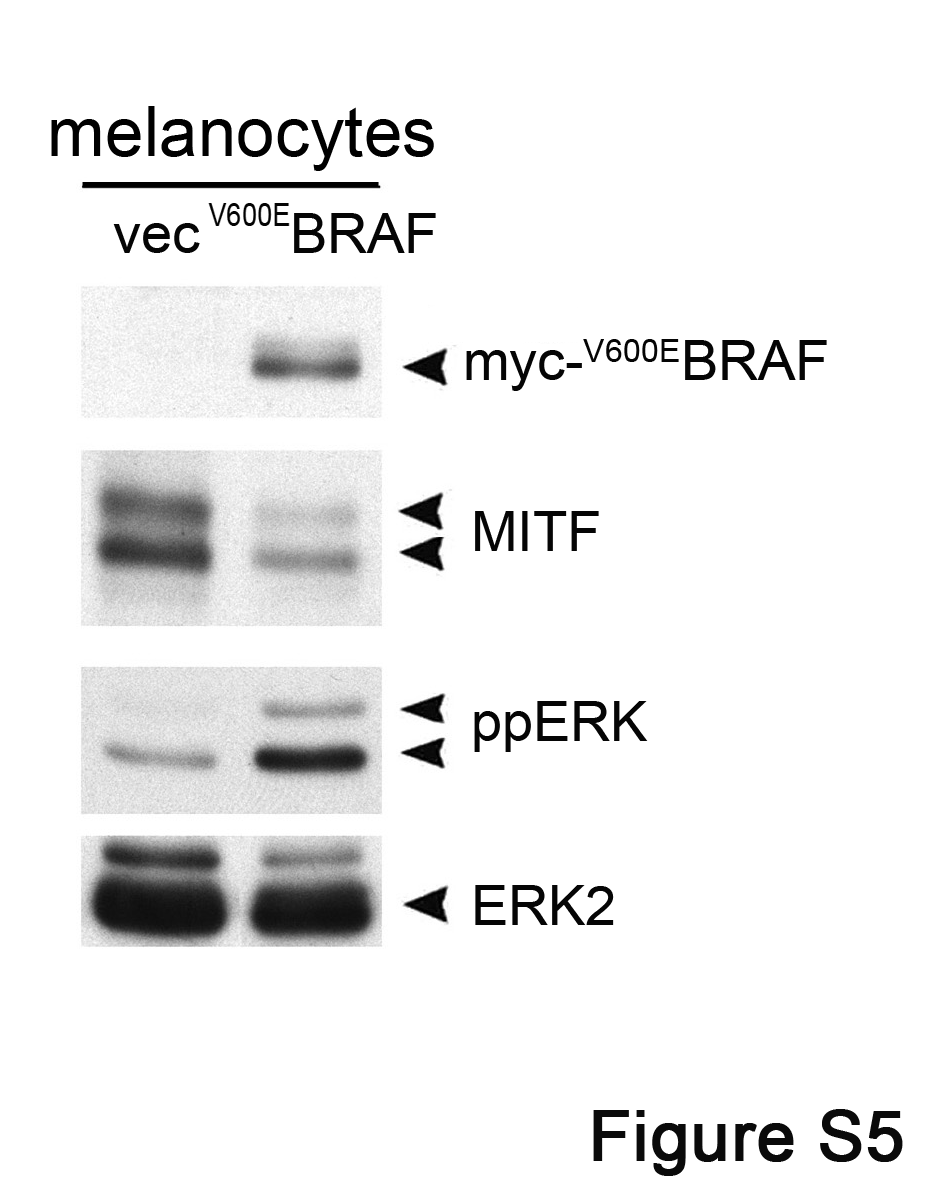

Supplement: Figure S5 — Oncogenic BRAF suppresses MITF protein expression. Oncogenic BRAF suppresses MITF protein levels. Western blot of human melanocytes transfected with V600EBRAF or an empty vector. Cells were analysed for myc-tagged V600EBRAF, MITF and ppERK. ERK2 was used as loading control. (0.21 MB TIF) [file pone.0002734.s005.tif]
